# How Visuals Affect Food Choice

**DOI:** 10.3390/foods9121835

**Published:** 2020-12-10

**Authors:** Iris Vermeir

**Affiliations:** BE4LIFE, Department of Economics and Business Administration, Ghent University, Tweekerkenstraat 2, 9000 Ghent, Belgium; Iris.Vermeir@UGent.be; Tel.: +32-9264-3524

In this Special Issue, we bring together nine original research articles that demonstrate how visual cues affect consumer reactions that drive food decisions. More specifically, these papers elaborate on the effects of (a combination of) external graphical elements of the packaging (e.g., labels, images), structural design elements of the packaging (e.g., package material) as well as spatial elements of the decision context (e.g., proximity) on a variety of perceptual (e.g., quality perception), attitudinal (e.g., product attitude) and behavioral (e.g., choice) outcomes. One review article digs deeper into why specific cues could influence consumer reactions and provides an overview of future research possibilities on the effects of visual cues on psychological processes and behavioral outcomes.

External graphical elements are for example package and label colors, type font of text on package or labels, images on packages or displays etc. In this Special Issue, Li and Dando [1] follow up on previous research showing that labels indicating healthy ingredients, origin or production methods trigger more positive sensory and nutritional expectations, possibly due to a positive halo effect. They test whether labels identical in image, font, color, and design but different in wording would affect liking of yoghurt. Interestingly, respondents liked yoghurt with identical sensorial characteristics less when it contained an “all-natural” claim than “high protein”, “low fat” or “with Stevia” claims demonstrating that labels can affect food preferences.

Similarly, Perez et al. [2] found that multiple product labels (containing information on geographical origin and production method) influence consumer choices for olive oil. Using a discrete choice experiment, they show that both labels on the geographic origin and organic production method render positive evaluations, but no major additional value lies in using both labels together. Consumers do seem to be willing to pay more for olive oil containing a geographical origin label than an organic product method label.

In a somewhat different context, Roose and Mulier [3] show that multisensory food ads only enhance taste perceptions of indulgent food but not for healthy food. An advertisement using images emphasizing multiple senses (e.g., taste, touch and smell) triggers sensory stimulation and positive sensory thoughts resulting in better taste perception than an advertisement emphasizing only taste. They argue that this is only the case for indulgent foods that are in need of multiple arguments persuading consumers to give in to this guilty pleasure. For healthy products, using multiple persuasive elements could trigger negative thoughts and lower taste perceptions.

Kunz et al. [4] assess whether the use of traffic light labeling could have unwanted side effects so that more healthy products are chosen less. They show in an online study that although traffic light labeling helps differentiate between healthy and less healthy alternatives (better than numerical ratings), it does not refrain people from making unhealthy choices. Extending previous research, they show that traffic light labeling did not affect the product’s expected tastiness expectations nor did it elicit reactance by a perceived threat of freedom. Traffic light labeling did increase purchase intentions for more healthy options while it did not affect purchase intentions of more unhealthy options. They conclude that a traffic light label might be used as a tool to assist consumers in making healthier food choices without taxing products’ desirability. They call for further research on moderating individual factors such as self-regulation and self-regulatory focus to assess the effect of traffic light labeling. Indeed, a lack of self-regulation can increase the effect of visual cues on consumption decisions and lead to overconsumption.

Szakaly et al. [5] test how people with presumably more and less self-regulation capacities react to different health-related labels. Health claims indicating a reduction in salt or a reduction in fat and salt content increased health perceptions but decreased taste expectations, purchase intentions and actual taste ratings. The authors discuss how a health halo or the automatic association of health-promoting products with less palatability and enjoyment (particularly in the case of “reduced” and “free from” version) could have distorted the actual perception of flavor.

Structural design elements are, for example, product or package material, shape or size. In this Special Issue, De Kerpel et al. [6] follow up on research suggesting a learned association between glossiness of package material and product greasiness arguing that a similar “glossy = sugary association” should also have been established since sugary products are often sold in glossy packages. They test whether the previously demonstrated association between glossiness and greasiness could be the result of an evolved, rather than a learned, association. A positive association between package glossiness and greasiness of the product might exist because fat and glossiness share some exterior resemblance rather than because of the often-used combination of glossy packaging and greasy products. Consistent with earlier research demonstrating that mate packaging affects perceived naturalness of the packaged product and perceived healthiness of the product, they found that health inferences were lower for glossy (versus mate) packages. Furthermore, quality and expensiveness ratings and even actual taste experiences were lower for chocolate packaged in glossy versus mate materials.

Spatial visual cues concern the location of objects, their movement and the spatial relation between an object and the self. Spatial elements entail for example the specific positioning of food on the shelf or menu (e.g. top or down position) or rotation of product presentation (e.g. right/left angle). In this Special Issue, Bischoff et al. [7] look at whether temporal distance moderates the effect that other people have on food choice and amount of food consumption. Social modelling of food intake has been demonstrated for different types of food, populations and eating contexts. People eat less when others eat a small amount and vice versa. Monitoring other people’s eating behavior could reduce our uncertainty on how much to eat or trigger automatic imitation of movements. Bischoff et al. investigate whether psychological (temporal) distance reduces the influence of observing other’s snacking behavior. Based on construal level theory, they hypothesize that mentally “zooming out” reduces imitation of the amount of food intake but not the imitation of contextually more invariant features such as the particular snack brand choice. Indeed, they found that participants primed with proximity imitated the model’s consumed quantity, whereas participants primed with distance did not, while no effects were found for brand choice.

On a more general level, Zhang et al. [8] postulate in this Special Issue that visual cues bias perceptions and behavior. Indeed, the awareness and understanding of sensory information (i.e., visual perception) is not always an accurate reflection of reality. Only those cues that are sufficiently salient are used to infer benefits (cfr. cue salience). Hence, different consumers may have varying and possibly conflicting perceptions of the same design, as they vary in which cues are salient to them in the first place. Our interpretation of visual cues can be biased, and these visual perception biases affect product judgments and actual consumption. Zhang et al. show that machines were better at classifying the origin of a dish based on visual properties, such as color brightness, saturation and texture, than human beings. Moreover, cultural and sociodemographic characteristics, as well as personal experience, affected accuracy ratings. They reason that humans probably derive expectations of taste or ingredients from visual cues which negatively affects accuracy of origin classification. Indeed, Lazard et al. state that our brains believe the reality of visual imagery (i.e., what we see is real) and claim future research is necessary on when we would or would not exert effort to disbelieve the interpretation we make from visual cues.

In this Special Issue, Chen et al. [9] dig deeper into how taste perception (sweetness) is perceived through sensory information coming from different senses. They use an immersive technology (Virtual Reality) to present visual cues to their participants to create a sense of immersion and feelings of presence and involvement. As visual cues, they use specific shapes, colors and textures that were either associated with a sweet taste or a bitter taste. In addition, they use both subjective self-reported hedonic ratings as well as two biometric measurements (Electroencephalogram and Facial Recognition) to measure positivity of reactions to the presented stimuli. Interestingly, visual–taste congruency affected an individual’s perception of sweetness but not product liking. The same beverage was rated significantly sweeter in a sweet-congruent environment than in an incongruent (bitter) or neutral environment but no differences in liking were noted. This is not in line with previous research suggesting that congruency of cross-modal correspondences may also affect people’s hedonic ratings.

Vermeir and Roose [10] propose future research to go beyond perceptual, attitudinal and behavioral measures by looking at psychological processes that could explain why specific visual cues affect consumer reactions. They provide an exemplary overview of psychological processes that could explain why specific visual cues affect consumer reactions at the point of purchase. They discern between object and spatial processed visual cues discussing effects of color, shape, aesthetic cues, materiality, text and picture combinations, location, movement and spatial relation between object and self. Using insights from several domains, they put forward how these specific visual cues can influence attention, cognitive, emotional and motivational reactions at the point of purchase, which in turn affects product perceptions, attitudes and behavioral outcomes. As such, Roose and Mulier already demonstrated that multisensory visual advertising cues affect cognitive reactions (positive, negative thoughts) and in turn affects taste expectations, product attitudes and purchase intentions. To guide future research, Vermeir and Roose sum up the most important research gaps in assessing whether and why visual cues affect food decisions at the point of purchase.

Overall, the work in this Special Issue provides examples of specific visual cues that can affect consumer outcomes. Several papers in this Special Issue demonstrate that particular visual cues such as labeling and package material affect consumer’s perceptions, attitudes and behavior. In addition, a future research agenda provides both researchers and practitioners with guidance to take on the challenge of understanding how visual cues can affect people’s food decisions at the point of purchase.
